# Body roundness index is a superior indicator to associate with the cardio‐metabolic risk: evidence from a cross‐sectional study with 17,000 Eastern-China adults

**DOI:** 10.1186/s12872-021-01905-x

**Published:** 2021-02-16

**Authors:** Jinjian Xu, Liqun Zhang, Qiong Wu, Yaohan Zhou, Ziqi Jin, Zhijian Li, Yimin Zhu

**Affiliations:** 1 Department of Epidemiology and Biostatistics, School of Public Health, Zhejiang University, Hangzhou, 310058 Zhejiang China; 2 Putuo District People’s Hospital, Zhoushan, 316100 Zhejiang China; 3 Department of Respiratory, Sir Run Run Shaw Hospital Affiliated to School of Medicine, Zhejiang University, Hangzhou, 310020 Zhejiang China; 4 The First Affiliated Hospital, School of Medicine, Zhejiang University, Hangzhou, 310058 Zhejiang China

**Keywords:** Body roundness index, Cardio‐metabolic risk factors, Eastern-China adults

## Abstract

**Background:**

To investigate the ability of body shape index (ABSI), body roundness index (BRI), waist circumference (WC), body mass index (BMI), waist-to-hip ratio (WHR), waist-to-height ratio (WHtR), and body adiposity index (BAI) for predicting non-adipose cardio-metabolic risk.

**Methods:**

A total of 17,360 Chinese subjects aged 18–95 years old who escaped cardiovascular disease (CVD) or diabetes were recruited in the cross-sectional study. Anthropometric and biochemical parameters were assessed. Receiver operating characteristic curve (ROC) and multinomial logistic regression were conducted to examine the association of anthropometric indicators with cardio-metabolic risk factors.

**Results:**

The mean age of subjects were 53.7(13.1) years, 41.6 % were males. The areas under the curve (AUC) demonstrated that WC, BMI, WHR, WHtR and BRI were able to predict high cardio-metabolic risk (AUC > 0.70). Meanwhile, multinomial logistic regression showed BRI was significantly associated with high cardio-metabolic risk (OR 3.27, 95% CI 3.01–3.55). The optimal cut-off values of BRI for high cardio-metabolic risk were (< 60 y: 3.49 vs. ≥60 y: 3.46) in males and (< 60 y: 3.47 vs. ≥60 y: 3.60) in females.

**Conclusions:**

WC, BMI WHR, and WHtR were potential obesity indicators in discriminating high cardio-metabolic risk, while BAI or ABSI was not. Moreover, BRI revealed superior predictive capacity and significant association with accumulated cardio-metabolic risk factors.

**Supplementary Information:**

The online version contains supplementary material available at 10.1186/s12872-021-01905-x.

## Background

Metabolic syndrome (MetSy) is a serious metabolic disorder consisting of obesity, hypertension, abnormal metabolism of lipoprotein and plasma glucose, which plays a vital role in the development of atherosclerotic heart disease, stroke and diabetes mellitus [1–3]. So far, over 33.9 % of older adults reached abnormal metabolism in China and caused a large disease-related healthcare burden [4–7].

MetSy was considered as an intermediate trait in the progression of severe cardiovascular disease (CVD) [8, 9] and can contribute to the risk of diabetes[10, 11]. However, obesity is an important component of metabolic syndrome that can independently contribute to the remaining components [12, 13]. Meanwhile, epidemiological studies have shown that obesity itself was a vital risk factor for hypertension [14], and cardiovascular disease [15] and diabetes [16]. Thus, that is necessary to explore the independent association between obesity and risk of CVD. Moreover, the remaining components of MetSy including hypertension, elevated fasting glucose (FPG), elevated triglycerides (TG), and reduced high-density lipoprotein cholesterol (HDL-C) were studied as the risks of CVD in previous studies [17–19]. Thus, the residual components of MetSy that excluding obesity were clustered as non-adipose cardio-metabolic risk factors will be more suitable for investigating the progress of cardiovascular disease or other adverse traits [4, 20]. Moreover, serum uric acid (SUA) is the end product of nucleic acid purine metabolism in body [21–24]. Previous studies revealed that an alteration of SUA level was associated with abnormal glucose and hyperuricemia, and it was considered to be a risk factor for MetSy or metabolic disorders [25–27].

Obesity was mainly due to the disproportionate growth of adipose tissue and lean body mass which can lead to further morbidity and mortality from cardiovascular disease [28, 29]. Recently, obesity was increasing rapidly worldwide and reached epidemic levels in China, which poses heavy public health and economic burdens [30]. The scanning of dual-energy X-ray absorptiometry (DXA), hydrostatic weighing, bioelectrical impedance and even skinfold thickness were considered as accurate methods for evaluating fat mass and distribution, whereas the use of such measurement was limited due to its complexity and/or cost [31, 32]. Epidemiological studies have revealed the anthropometric measures were acceptable indicators in evaluating obesity status for their simplicity and usefulness [20, 33–35]. The body mass index (BMI) has been considered as a diagnostic index for general obesity and could reflect the overall distribution of body fat [36, 37]. Meanwhile, waist circumference (WC), body adiposity index (BAI), waist-to-hip ratio (WHR) and waist-to-height ratio (WHtR) were studied to predict metabolic risk in previous studies [5, 38–40]. However, the traditional anthropometric indices failed to discriminate between fat and muscle mass [41, 42]. Recently, body shape index (ABSI) which reflects the body shape using waist circumference (WC), weight, and height and body roundness index (BRI) combines height and WC to predict the percentage of total and regional fat have been developed to overcome the limits of traditional obesity markers [33, 43–49]. A previous study found the two new indices were associated with abdominal adipose and more associated with cardiometabolic risk, onset of diabetes and premature mortality hazards than BMI and WC [4, 50, 51]. However, few shreds of evidence compared ABSI, BRI with common anthropometric indicators such as WC in the assessment of cardiometabolic risk factors in Chinese.

In this study, we clustered elevated blood pressure (BP), elevated FPG, elevated TG, reduced HDL-C and elevated serum uric acid (SUA) as non-adipose cardio-metabolic risk factors and adopted seven anthropometric indices including waist circumference (WC), body mass index (BMI), waist-to-hip ratio (WHR), waist-to-height ratio (WHtR), body adiposity index (BAI), body shape index (ABSI) and body roundness index (BRI) to conduct a population-based cross-sectional study to assess the ability of obesity indicators for predicting non-adipose cardio-metabolic risk and explore the pre-cardiovascular status in Chinese adults.

## Methods

### Study population

**Fig. 1:**
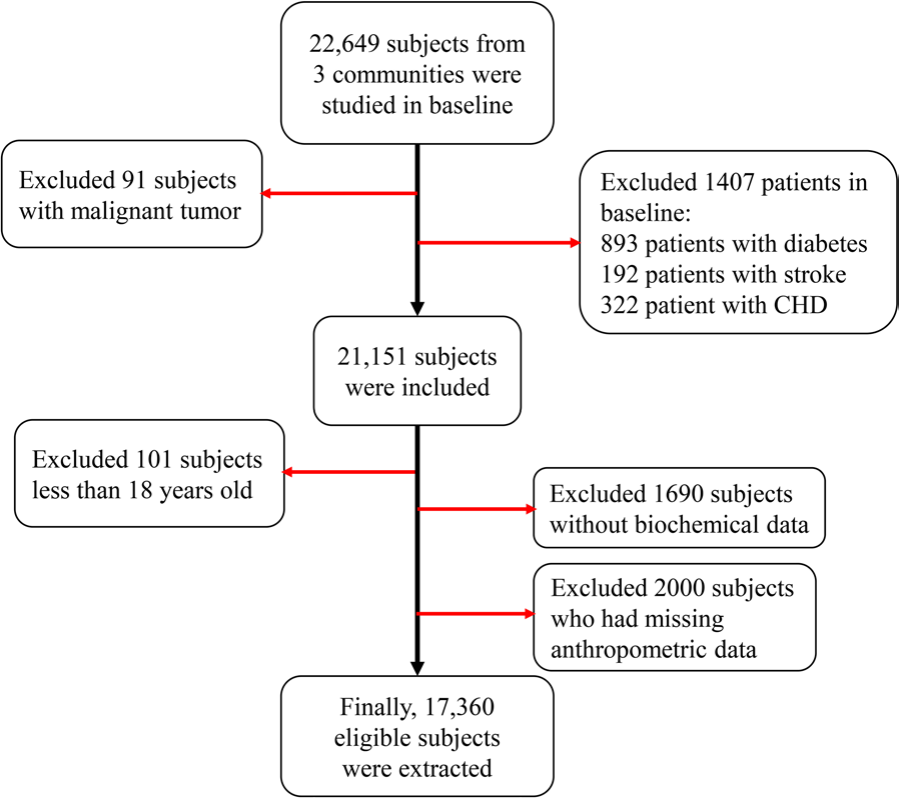
Flowchart of inclusion criteria for participants in this study

The cross-sectional data sources were the baseline records from 2009 to 2012 in Zhejiang Metabolic Syndrome Cohort (ZJMSC). ZJMSC is an ongoing community-based longitudinal study initiated in 2009 years included four communities and consisted of 22,649 participants aged 18–75 years old who have lived in Zhejiang province (Hangzhou, Zhoushan, Jiaxing, Jinhua) for more than 5 years and the follow-up visit was held from 2009 to 2019. A total of 22,649 participants (9,527 males and 13,122 females, mean age were 54.86 ± 14.2 years old) were recruited in the present study. Subjects were defined as the young group (< 60 years old) and the older group (≥ 60 years old). Exclusion criteria: (1) severe infective diseases and malignancies in baseline; (2) patients with type 2 diabetes mellitus (T2DM), cardiovascular disease (CVD) and stroke in baseline; (3) less than 18 years old; (4) without biochemical and anthropometric data. A total of 17,360 (7,226 males and 10,134 females) participants met these criteria and were finally included in this study. Additionally, inclusion criteria and selection flowchart for all samples as shown in Fig. 1. This study was approved by the Human Research Ethics Committee of Zhejiang University, Zhejiang, China. All participants provided written informed consent before participation.

### Measurements

#### Anthropometric variables and covariates

For each participant, covariates of demographic characteristics, smoking and alcohol drinking status, diet, and physical activity was collected with questionnaire-based interview. In the present study, smoking and drinking were categorized as current, former and never. Current smokers and drinkers were defined as those who were still smoking or drinking during the investigation. Former smokers and drinkers were those who had a history of smoking or drinking and stopped for at least one year. The occupational labor strength was defined as mild labor, moderate labor, and heavy labor.

Height (cm), body weight (kg), waist circumference (WC; cm), hip circumference (HC; cm), systolic blood pressure (SBP) and diastolic blood pressure (DBP) were measured by trained assistants using medical equipment. Height and weight were measured with the subjects wearing light clothing and no shoes. Height was recorded to the nearest 0.1 cm and body weight to the nearest 0.1kg. Body mass index (BMI) was computed as weight (kg) divided by height squared (m^2^). The girth of the midpoint between the lowest point of the rib and the upper edge of the iliac crest were calculated as waist circumference (WC). The length of the horizontal position of the hip protrusion was calculated as the hip circumference (HC) [52]. The measurements of WC and HC were taken to the nearest 0.1 cm. Waist-to-hip ratio (WHR) and waist-to-height ratio (WHtR) were respectively calculated as WC divided by HC and WC divided by height. BAI was calculated as HC (cm)/height^1.5^ (m) minus 18 [53]. ABSI was calculated using the formula as $\tt \text{ABSI}=\frac{\hbox{WC}}{{{\hbox{BMI}}^{2/3}{Height}}^{1/2}}$ and BRI was calculated using the formula as $\hbox{BRI}=364.2-365.5\times \sqrt{1-\left(\frac{(\hbox{WC}/(2{\uppi }{\left)\right)}^{2}}{(0.5\,\hbox{Height}{)}^{2}}\right)}$. Sitting blood pressure was measured 2 times after at least 10 minutes of rest using the standardized desktop sphygmomanometer. The average blood pressure derived from two measurement readings was used [43, 51]. All variables were collected according to standard interview guidelines and standard protocols [54–57].

#### Biochemical measurements

After an overnight fast of at least 12 hours, intravenous blood samples of each participant were collected, with one tube for Ethylene Diamine Tetraacetic Acid (EDTA) anticoagulation and one tube for separation gel coagulation, then centrifuged at 4000 rpm for 15 min at 4℃, the aliquots were stored at − 80℃ until use. Fasting plasma glucose (FPG) was measured by the hexokinase method, SUA and lipid profile containing total cholesterol (TC), triglycerides (TG), high-density lipoprotein cholesterol (HDL-C) and low-density lipoprotein cholesterol (LDL-C) were measured by the enzymatic method using an Abbott Aeroset autoanalyzer. Alanine transaminase (ALT) and aspartate transaminase (AST) were measured with a rating method. Blood urea nitrogen was measured with uricase ultraviolet method and serum creatinine with a picric acid method.

### Criteria for cardio‐metabolic risk factors

According to the diagnosis criteria of MetSy by the International Diabetes Federation (IDF) in 2009 [58], the comprehensive non-adipose cardiometabolic risk factors included the following items: (1) elevated BP: systolic blood pressure (SBP) (≥ 130 mmHg) or diastolic blood pressure (DBP) (≥ 85 mm Hg), or ongoing antihypertensive medications; (2) elevated FPG: FPG ≥ 5.6 mmol/L and/or diagnosis of type 2 diabetes, or ongoing anti-diabetic treatment; (3) elevated TG: TG ≥ 1.7 mmol/L; (4) reduced HDL-C: HDL-C < 1.03 mmol/L in men and HDL-C < 1.29 mmol/L in women. In addition, we also enrolled: (5) elevated SUA: SUA > 420µmol/L according to the diagnosis criteria of hyperuricemia in 2000 [59]. The people with three or more risks were diagnosed as “high cardio-metabolic risk population” in present study.

### Statistics

The continuous variables were presented as means and standard deviations (SD), categorical variables as counts or percentages. The one-way analysis of variance and the chi-squared test were used to compare the baseline characteristics of participants stratified by the number of risk factors. The linear correlation was conducted to explore the relationship among multiple anthropometric indicators and revealed the mutative trend between median of anthropometric indicators and the number of cardiometabolic risk factors. In addition, multiple comparisons were conducted between the highest risk group and residual groups. Adjusted multinomial logistic regression was conducted to examine the associations of adiposity indicators with cardio-metabolic risk. To determine the optimal cut-off values and to compare the ability of the obesity indices for predicting multiple metabolic risk factors, the receiver operating characteristic curve (ROC) and area under the curve (AUC) were performed and optimal cut-off values were identified from the maximum Youden index (sensitivity plus specificity-1). The Hanley and McNeil method was used to compare the inter-group differences of AUCs. The analysis was performed by SPSS 25.0 software. All analyses were two-sided, and the difference was statistically significant at *P* < 0.05.

## Results

### Basic characteristics of the objects

According to the number of cardio-metabolic risk factors, subjects were divided into non-metabolic risk group, low metabolic risk group (< 3) and high metabolic risk group (≥ 3), and the differences of basic characteristics were analyzed by the presence of cardio-metabolic risk factors (Table 1). The proportion of females was higher in high metabolic risk groups (58.7 %). Participants with high cardio-metabolic risk were older than those with none or low metabolic risk (*P* < 0.001). In addition, participants with high cardio-metabolic risk were more likely to have higher values of SBP, DBP, FPG, TG, HDL-C, LDL-C, and SUA and lower levels of HDL-C than the other groups (*P* < 0.001). Moreover, participants with high cardio-metabolic risk were more likely to have higher BMI, WC, HC, WHR, WHtR, BAI, ABSI and BRI (*P* < 0.001). In addition, the average values of SBP, FPG and TG were abnormal in high cardio-metabolic risk group, which were 146.58 (19.23) mmHg, 5.58 (1.52) mmol/L and 2.91 (2.04) mmol/L, respectively.

**Table 1:** Baseline characteristics of study subjects

Variables	N	Number of cardio-metabolic abnormalities			*P* value
		None	< 3	≥ 3	
Sex, N (%)					< 0.001
Male	7226	1785 (38.3 %)	4308 (43.3 %)	1133 (41.3 %)	
Female	10,134	2876 (61.7 %)	5650 (56.7 %)	1608 (58.7)	
Age (years)	17,290	48.44 (14.88)	56.63 (14.11)	59.66 (12.57)	< 0.001
Smoker, N (%)	3575	923 (21.21 %)	2099 (22.35 %)	553 (21.63 %)	0.295
Alcohol drinker, N (%)	4191	1098 (26.94 %)	2438 (27.66 %)	655 (26.57 %)	0.471
Occupational labor strength, N (%)					< 0.001
Mild	10,040	2444 (57.93 %)	5726 (62.73 %)	1870 (72.12 %)	
Moderate	3049	958 (22.71 %)	1754 (19.22 %)	337 (13.00 %)	
Heavy	2851	817 (19.36 %)	1648 (18.05 %)	386 (14.89 %)	
Height (cm)	17,057	160.69 (7.95)	160.05 (8.33)	159.8 (8.57)	< 0.001
Weight (kg)	16,995	55.91 (8.67)	59.48 (10.29)	64.2 (10.75)	< 0.001
WC (cm)	17,018	75.11 (8.36)	80.27 (9.16)	85.8 (8.6)	< 0.001
HC (cm)	17,022	90.13 (6.09)	92.11 (6.71)	94.72 (6.7)	< 0.001
BMI (kg/m^2^)	16,967	21.62 (2.79)	23.15 (3.16)	25.06 (3.14)	< 0.001
WHR	17,004	0.83 (0.07)	0.87 (0.07)	0.91 (0.06)	< 0.001
WHtR	16,961	0.47 (0.05)	0.5 (0.06)	0.54 (0.05)	< 0.001
BAI	16,965	26.4 (3.95)	27.65 (4.15)	29.07 (4.29)	< 0.001
ABSI	16,878	0.08 (0.005)	0.08 (0.005)	0.08 (0.005)	< 0.001
BRI	16,961	2.83 (0.95)	3.47 (1.12)	4.15 (1.12)	< 0.001
ALT (U/L)	11,645	20.00 (13.92)	23.01 (16.79)	28.59 (20.66)	< 0.001
AST (U/L)	9354	24.99 (10.71)	27.45 (14.65)	29.85 (16.36)	< 0.001
Serum creatinine (umol/L)	9615	89.22 (12.80)	91.12 (16.81)	93.94 (17.71)	< 0.001
Blood urea nitrogen (mmol/L)	9358	5.6 (1.61)	5.86 (2.86)	5.71 (1.66)	< 0.001
SBP (mmHg)	17,359	112.52 (10.2)	133.94 (21.15)	146.58 (19.23)	< 0.001
DBP (mmHg)	17,359	68.97 (7.85)	79.01 (11.65)	84.64 (11.1)	< 0.001
FPG (mmol/L)	17,360	4.6 (0.48)	4.93 (1)	5.58 (1.52)	< 0.001
TG (mmol/L)	17,360	0.97 (0.32)	1.52 (1.04)	2.91 (2.04)	< 0.001
HDL–C(mmol/L)	17,360	1.62 (0.33)	1.5 (4.4)	1.16 (0.31)	< 0.001
LDL–C(mmol/L)	17,344	2.52 (0.73)	2.76 (0.79)	2.88 (0.84)	< 0.001
SUA (µmol/L)	17,360	280.96 (65.57)	315.93 (87.93)	377.27 (109.3)	< 0.001
Elevated BP	8578	0	6076 (61.0 %)	2502 (91.3 %)	< 0.001
Elevated FPG	2574	0	1374 (13.8 %)	1200 (43.8 %)	< 0.001
Elevated TG	5134	0	2793 (28.0 %)	2341 (85.4 %)	< 0.001
Reduced HDL-C	4506	0	2651 (26.6 %)	1855 (67.7 %)	< 0.001
Elevated SUA	2222	0	1196 (12.0 %)	1026 (37.4 %)	< 0.001

Data are expressed as mean ± standard deviation or counts (percentages)

*WC* waist circumference, *HC* hip circumference, *BMI* body mass index, *WHR* waist-to-hip ratio, *WHtR* waist-to-height ratio, *BAI* body adiposity index, *ABSI* a body shape index, *BRI* body roundness index, *SBP* systolic blood pressure, *DBP* diastolic blood pressure, *FPG* fasting plasma glucose, *TG* triglyceride, *HDL-C* high-density lipoprotein cholesterol, *LDL-C* low-density lipoprotein cholesterol, *SUA* serum uric acid, *BP* blood pressure, *ALT* alanine transaminase, *AST* aspartate transaminase

### Cardio‐metabolic risk factors and anthropometric indicators

Table 2 shows the mean value of anthropometric parameters in multiple groups by the presence of cardio-metabolic factors. WC, BMI, WHR, WHtR, BAI, ABSI and BRI increased significantly with the number of cardio-metabolic risk factors (*P*_trend < 0.01). Moreover, the averages of indicators in the highest cardio-metabolic risk group were greater than residual groups (*P* < 0.01), and the values of WC, BMI, WHR, WHtR, BAI, ABSI and BRI were 87.25 cm, 26.68 kg/m^2^, 0.94, 0.54, 26.98, 0.078 and 4.23 in males, in females were 88.69 cm, 26.04 kg/m^2^, 0.92, 0.58, 32.31, 0.081 and 4.97. Meanwhile, the correlation matrix between anthropometric indicators was presented in Additional file 1: Table S1. In both men and women, BRI showed a strong correlation with WC (men: r = 0.93, *P* < 0.01; women: r = 0.94, *P* < 0.01) and WHtR (men: r = 0.99, *P* < 0.01; women: r = 0.99, *P* < 0.01), while the linear correlation between BRI and BMI (men: r = 0.80, *P* < 0.01; women: r = 0.77, *P* < 0.01) was relatively weak (Additional file 1: Table S1, Additional file 2: Additional Fig. S1a, b).

**Table 2:** The alteration of anthropometric indicators with the increase of cardiometabolic factors

Variables	Number of cardiometabolic risk factors						
	0	1	2	3	4	5	*P_*trend
Male	1742	2400	1690	810	250	28	
WC	77.17 ± 7.94**	80.52 ± 8.48**	83.73 ± 8.91**	87.45 ± 8.48**	89.15 ± 7.42*	89.25 ± 8.79	< 0.001
BMI	21.59 ± 2.64**	22.71 ± 2.91**	23.87 ± 3.06**	25.08 ± 3.12**	25.60 ± 2.84**	26.68 ± 3.26	< 0.001
WHR	0.85 ± 0.06**	0.88 ± 0.06**	0.90 ± 0.06**	0.92 ± 0.06**	0.93 ± 0.05**	0.94 ± 0.05	< 0.001
WHtR	0.46 ± 0.05**	0.48 ± 0.05**	0.50 ± 0.05**	0.52 ± 0.05**	0.54 ± 0.05*	0.54 ± 0.06	< 0.001
BAI	23.98 ± 3.08**	24.89 ± 3.27**	25.72 ± 3.23**	26.28 ± 3.24*	26.75 ± 3.47*	26.98 ± 5.39	< 0.01
ABSI	0.077 ± 0.005**	0.078 ± 0.005*	0.079 ± 0.005*	0.079 ± 0.004**	0.080 ± 0.004**	0.078 ± 0.006	< 0.01
BRI	2.73 ± 0.84**	3.13 ± 0.95**	3.49 ± 0.99**	3.87 ± 0.99**	4.10 ± 0.93**	4.23 ± 1.26	< 0.001
Female	2848	3216	2282	1228	306	28	
WC	73.81 ± 8.37**	77.68 ± 9.06**	81.14 ± 9.13**	83.91 ± 8.44**	86.29 ± 8.26**	88.69 ± 7.16	< 0.001
BMI	21.67 ± 2.88**	22.75 ± 3.12**	23.87 ± 3.30**	24.79 ± 3.08**	25.73 ± 3.30**	26.04 ± 2.88	< 0.001
WHR	0.82 ± 0.07**	0.85 ± 0.07**	0.87 ± 0.07**	0.89 ± 0.07**	0.90 ± 0.06**	0.92 ± 0.06	< 0.001
WHtR	0.47 ± 0.06**	0.50 ± 0.06**	0.52 ± 0.06**	0.54 ± 0.06**	0.56 ± 0.06**	0.58 ± 0.06	< 0.001
BAI	27.92 ± 3.65**	29.08 ± 3.67**	30.03 ± 3.89**	30.67 ± 3.77**	31.60 ± 3.90**	32.31 ± 4.79	< 0.01
ABSI	0.076 ± 0.006**	0.078 ± 0.006**	0.079 ± 0.006**	0.079 ± 0.005**	0.080 ± 0.005*	0.081 ± 0.005	< 0.01
BRI	2.89 ± 1.01**	3.42 ± 1.14**	3.86 ± 1.20**	4.22 ± 1.14**	4.55 ± 1.17**	4.97 ± 1.33	< 0.001

Data are expressed as mean ± standard deviation.

*WC* waist circumference, *BMI* body mass index, *WHR* waist-to-hip ratio, *WHtR* waist-to-height ratio, *BAI* body adiposity index, *ABSI* a body shape index, *BRI* body roundness index, *P*_trend: the median of anthropometric indicators increased significantly with the number of cardiometabolic risk factors. The statistic significances between the highest risk group and other groups are presented by * and **, *P < 0.01; ** *P* < 0.001

### AUCs of anthropometric indicators for cardio‐metabolic risk factors

Based on the result of ROC, the AUCs for each anthropometric indicator to predict multiple cardio-metabolic risk factors were presented in Additional file 1: Table S2. The AUCs presented that WC, BMI, WHR, WHtR, and BRI had better predictive ability for elevated TG in males and their AUCs were 0.70, 0.70, 0.68, 0.70 and 0.70. Similarly, the superior abilities of WC, BMI, WHR, WHtR and BRI to predict TG were observed in female, the AUCs were 0.69, 0.68, 0.67, 0.69 and 0.69. In addition, WC, WHtR and BRI displayed a superior predictive ability to high SUA in females, with AUCs of 0.69, 0.70 and 0.70, respectively. Moreover, the AUCs of obesity parameters for predicting cardio-metabolic risk factors were significant (Additional file 1: Table S2, Additional file 3: Fig. S2a–j).

**Fig. 2:**
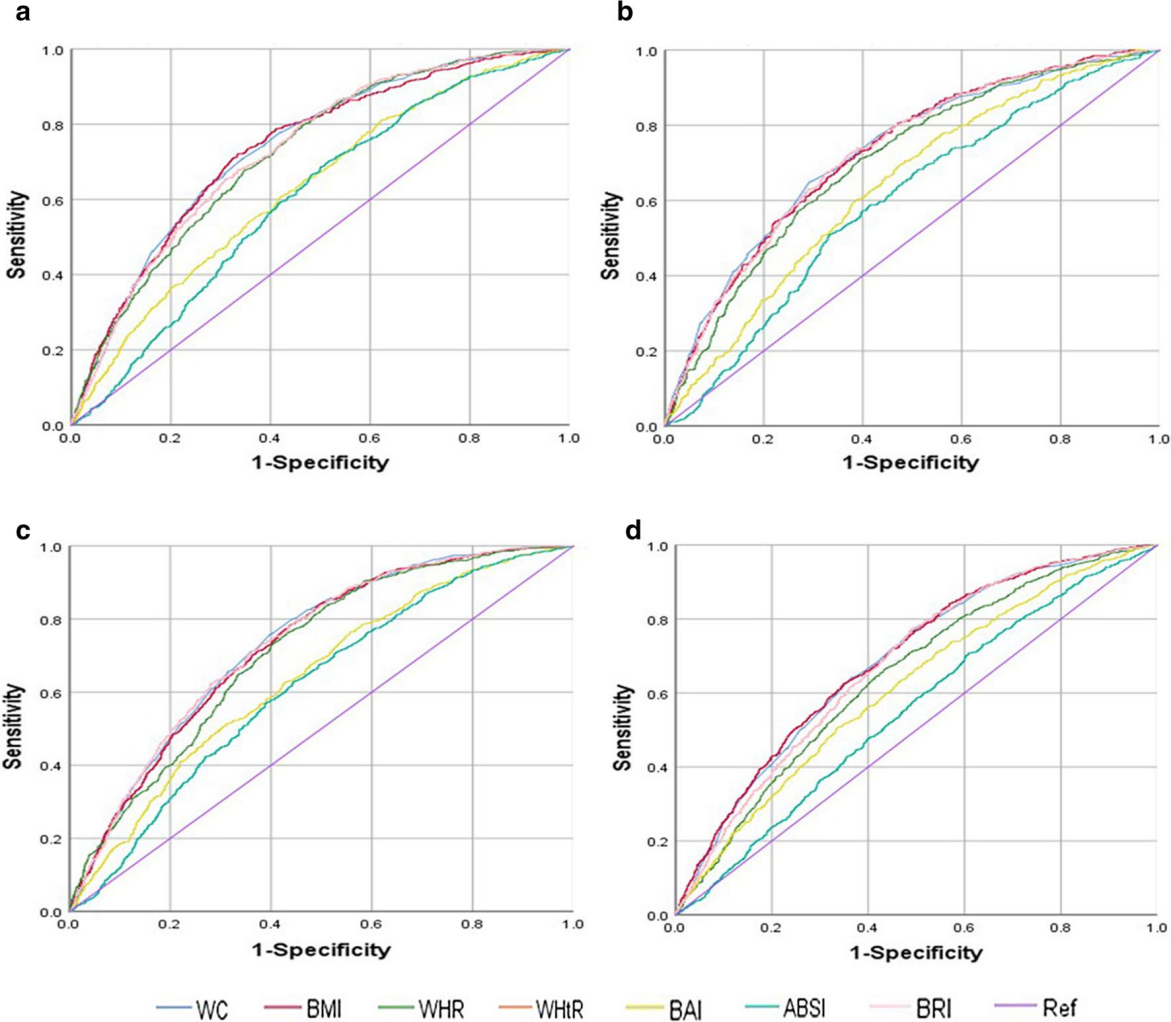
ROC curves of anthropometric indicators to predict high cardiometabolic risk. **a** ROC curves in young (< 60 years old) group of males. **b** ROC curves in older (≥ 60 years old) group of males. **c** ROC curves in young (< 60 years old) group of females. **d** ROC curves in older (≥ 60 years old) group of females

Table 3 shows that WC, BMI, WHR, WHtR and BRI revealed similar abilities to predict high cardio-metabolic risk. In the young group (< 60 years), the AUCs for males were 0.74, 0.74, 0.73, 0.73, 0.73, for females were 0.73, 0.73, 0.71, 0.73 and 0.73. However, in older group (≥ 60 years), the AUCs of WC, BMI, WHR, WHtR and BRI were 0.73, 0.73, 0.70, 0.73 and 0.73 for males, and were 0.69, 0.70, 0.65, 68 and 0.68 for females. In addition, the best cut-off values of WC, BMI, WHR, WHtR and BRI were 84.45 cm, 24.04 kg/m^2^, 0.88, 0.51 and 3.49 in males within young group (< 60 years) and in older group (≥ 60 years), the best cut-off values were 84.75 cm, 23.24 kg/m^2^, 0.90, 0.51 and 3.46. Meanwhile, in females, the best cut-off values of WC, BMI, WHR, WHtR and BRI were 78.90 cm, 22.94 kg/m^2^, 0.85, 0.51 and 3.47 within young group (< 60 years) and were 78.85 cm, 23.73 kg/m^2^, 0.88, 0.51 and 3.60 in older group (≥ 60 years). Meanwhile, the sensitivity, specificity and Youden index of anthropometric measurements to predict high cardio-metabolic risk was shown in Table 3. In addition, the differences of predictive ability for high cardio-metabolic risk factors by anthropometric indicators were significant (P < 0.001) (Table 3; Fig. 2a–d).

**Table 3:** AUC, optimal cut-off values, sensitivity, specificity and Youden index of anthropometric indicators to predict high cardio-metabolic risk

	< 60 years					≥ 60 years				
	AUC (95 %CI)	Cut-off value	Sensitivity	Specificity	Youden index	AUC (95 %CI)	Cut-off value	Sensitivity	Specificity	Youden index
*Male*										
WC	0.74 (0.72–0.76)**	84.45	0.71	0.66	0.36	0.73 (0.70–0.75)**	84.75	0.65	0.71	0.36
BMI	0.74 (0.72–0.76)**	24.04	0.72	0.67	0.39	0.73 (0.70–0.75)**	23.24	0.70	0.63	0.34
WHR	0.73 (0.71–0.75)**	0.88	0.80	0.54	0.33	0.70 (0.68–0.73)**	0.90	0.71	0.61	0.32
WHtR	0.73 (0.71–0.75)**	0.51	0.65	0.69	0.34	0.73 (0.70–0.75)**	0.51	0.67	0.67	0.35
BAI	0.63 (0.61–0.66)**	25.12	0.61	0.58	0.19	0.64 (0.61–0.67)**	25.24	0.69	0.53	0.22
ABSI	0.61 (0.58–0.63)**	0.08	0.68	0.50	0.19	0.60 (0.57–0.62)**	0.08	0.51	0.67	0.18
BRI	0.73 (0.71–0.75)**	3.49	0.65	0.69	0.34	0.73 (0.70–0.75)**	3.46	0.72	0.63	0.35
*Female*										
WC	0.73 (0.71–0.75)**	78.90	0.76	0.60	0.36	0.69 (0.67–0.71)**	78.85	0.77	0.51	0.28
BMI	0.73 (0.71–0.75)**	22.94	0.78	0.56	0.35	0.70 (0.67–0.71)**	23.73	0.62	0.65	0.28
WHR	0.71 (0.69–0.73)**	0.85	0.73	0.60	0.33	0.65 (0.63–0.67)**	0.88	0.66	0.57	0.23
WHtR	0.73 (0.71–0.75)**	0.51	0.71	0.64	0.35	0.68 (0.66–0.70)**	0.51	0.77	0.51	0.28
BAI	0.64 (0.62–0.66)**	27.89	0.76	0.44	0.21	0.61 (0.59–0.64)**	30.99	0.51	0.67	0.17
ABSI	0.62 (0.59–0.64)**	0.0763	0.63	0.56	0.18	0.55 (0.53–0.57)**	0.0781	0.71	0.39	0.10
BRI	0.73 (0.71–0.75)**	3.47	0.71	0.64	0.35	0.68 (0.66–0.70)**	3.60	0.77	0.51	0.28

**P value < 0.001: the inter-group difference of AUCs under the ROC curve; high cardio-metabolic risk: greater than or equal to three risk factors

### Associations of anthropometric indicators with cardio‐metabolic risk factors

The results of logistic regression revealed that WC, BMI, WHR, WHtR and BAI, ABSI and BRI were significantly associated with accumulated cardio-metabolic risk factors in model 1 (Table 4). After adjusted covariates, the association of ABSI with high cardio-metabolic risk was suppressed (OR 1.08, 95% CI 1.01–1.16). Meanwhile, WC (OR 1.16, 95% CI 1.15–1.17), BMI (OR 1.45, 95% CI 1.41–1.47), WHR (OR 1.79, 95% CI 1.74–1.83), WHtR (OR 1.76, 95% CI 1.67–1.85) and BAI (OR 1.23, 95% CI 1.21–1.26) were associated with high accumulated cardio-metabolic risk in model 3. Moreover, BRI demonstrated the strongest relationship to accumulated cardio-metabolic risk regardless of lifestyles (OR 3.27, 95% CI 3.01–3.55).

**Table 4:** Association of anthropometric indicators with accumulated cardio-metabolic risk factors

Number of cardio-metabolic risk factors	Anthropometric indicators						
	WC	BMI	WHR	WHtR	BAI	ABSI	BRI
*Unadjusted*	OR (95 % CI)	OR (95 % CI)	OR (95 % CI)	OR (95 % CI)	OR (95 % CI)	OR (95 % CI)	OR (95 % CI)
None	Ref	Ref	Ref	Ref	Ref	Ref	Ref
1	1.05 (1.05–1.06)	1.14 (1.13–1.16)	1.37 (1.25–1.49)	1.09 (1.04–1.13)	1.06 (1.05–1.07)	1.08 (1.03–1.13)	1.66 (1.59–1.73)
2	1.10 (1.09–1.10)	1.29 (1.27–1.31)	1.62 (1.56–1.69)	1.41 (1.18–1.65)	1.12 (1.10–1.13)	1.13 (1.06–1.20)	2.36 (2.26–2.47)
≥ 3	1.15 (1.14–1.15)	1.45 (1.42–1.47)	1.77 (1.71–1.83)	1.80 (1.74–1.87)	1.17 (1.16–1.18)	1.49 (1.32–1.66)	3.21 (3.05–3.38)
*Model 1*							
None	Ref	Ref	Ref	Ref	Ref	Ref	Ref
1	1.05 (1.04–1.05)	1.15 (1.14–1.17)	1.38 (1.34–1.41)	1.10 (1.00–1.21)	1.07 (1.06–1.09)	0.86 (0.70–1.02)	1.53 (1.46–1.59)
2	1.09 (1.09–1.10)	1.31 (1.29–1.33)	1.61 (1.57–1.64)	1.39 (1.30–1.48)	1.15 (1.13–1.16)	1.30 (0.90–1.70)	2.17 (2.07–2.27)
≥ 3	1.14 (1.14–1.15)	1.48 (1.46–1.51)	1.91 (1.88–1.94)	1.56 (1.45–1.66)	1.21 (1.19–1.23)	1.37 (0.82–1.93)	2.94 (2.78–3.09)
*Model 2*							
None	Ref	Ref	Ref	Ref	Ref	Ref	Ref
1	1.06 (1.05–1.06)	1.16 (1.13–1.18)	1.37 (1.31–1.43)	1.20 (1.11–1.29)	1.10 (1.08–1.12)	0.63 (0.23–1.03)	1.67 (1.56–1.80)
2	1.10 (1.10–1.11)	1.29 (1.26–1.32)	1.60 (1.54–1.66)	1.44 (1.39–1.50)	1.17 (1.15–1.20)	1.02 (0.98–1.06)	2.38 (2.20–2.57)
≥ 3	1.16 (1.15–1.17)	1.45 (1.41–1.49)	1.79 (1.74–1.83)	1.76 (1.67–1.85)	1.23 (1.21–1.26)	1.08 (1.01–1.16)	3.27 (3.01–3.55)

Data are shown as OR (95 % CIs). The ORs describe the association with increased cardio-metabolic risk factors refer to without cardio-metabolic risk factor group; Model 1: adjusted for age, sex; Model 2: adjusted for model 1 + current smoking, alcohol use, physical activity, alanine transaminase, aspartate transaminase, serum creatinine, blood urea nitrogen

## Discussion

The present results indicated that WC, BMI, WHR, WHtR and BRI were able to similarly predict high cardio-metabolic risk, while BAI or ABSI was not. Moreover, BRI revealed specific predictive ability for elevated TG in the overall population and for elevated SUA in females. The superior predictive capacities and significant associations between BRI and high cardio-metabolic risk were found after adjusted covariates.

Body fat attracted intense attention when it linked with the development of cardiovascular disease and other adverse events [39, 60]. Generally, the adipose tissue concentrate in the abdomen could produce various compounds of autocrine, paracrine and endocrine activities, which could influence the metabolism and cardiovascular system [61–64]. Obesity-related indicators are non-invasive for predicting body fat mass which will become the simple and practical adiposity markers[65]. The traditional indicators of WC, BMI, WHR, WHtR and BAI were widely recruited to explore the association of adiposity with metabolic disorder, cardiovascular morbidity and diabetes, but the results were unclear due to the delusive relationship with fat mass and distribution [44, 66–68]. Meanwhile, ABSI and BRI were considered as effective indicators for predicting obesity status and risk of metabolic syndrome [51, 69]. A previous study examined the predictive capacity of ABSI and BRI to identify hyperuricemia and to compare the relative strength of association between anthropometric indices and hyperuricemia in rural China [51]. The results found BRI (AUC: 0.641; OR: 1.459) showed more powerful predictive ability for hyperuricemia than BMI (AUC: 0.630; OR: 1.108), while having a similar predictive power as WHtR (AUC: 0.656; OR:1.067) and WC (AUC: 0.658; OR: 1.047) in the female group, but not in the male group. However, ABSI had the lowest predictive power for hyperuricemia in both sex categories. Based on the large-scale population, the similar results were stably validated in our study and the predictive AUC for hyperuricemia was 0.70 in females. For the predictive capacity and association to cardiometabolic risk factors, BRI could be used as an alternative body index to WHtR, while ABSI could not. Meanwhile, a study with Polish population also showed that BRI had superior ability to predict metabolic syndrome [46]. Generally, BRI possesses a stable ability to predict cardiometabolic risk and metabolic syndrome even in different ethnic groups, and these results were well determined in our study [49]. Moreover, Chang et al. [64]. found that BRI (AUC = 0.74), not ABSI was superior measurement compared to BMI, WC and WHtR for determining the presence of left ventricular hypertrophy, especially for eccentric left ventricular hypertrophy [50]. Obesity was supposed to increase fat accumulation in the cardiac which is associated with ventricular hypertrophy and ventricular systolic dysfunction [70]. Body roundness index (BRI) was developed to predict both body fat and the percentage of visceral adipose tissue using WC in relation to height, which will estimate the shape of the human body figure as an ellipse [4, 43, 71]. BRI was generally used as an adipose indicator for determining the presence of hyperuricemia, arterial stiffness, CVD, diabetes, dyslipidemia, hypertension, and MetSy [7, 46, 49, 51, 67, 68]. However, BRI was limited in predicting percentage of fat mass in athletes when compared with bio-impedance analysis or skinfold prediction [72]. Although BRI was not superior to WHtR for determining the presence of MetSy or cardio-metabolic risk, there were no significant differences in predicting MetS, suggesting that BRI was an alternative obesity indicator [43, 73]. The advantages of BRI exceed BMI and WHR were commonly believed to improve the predictive power of body fat and visceral adipose tissue and visceral adipose tissue was associated with MetSy was well known [46, 50, 74].

The present study included the large sample size and involved multi-regional population, which avoided the shortcomings of many previous studies. We confirmed BRI was superior indicator for multiple cardio-metabolic risk factors in Chinese adults. Meanwhile, a cross-sectional study also revealed only BRI, not ABSI, can determine the presence of MetSy and insulin resistance (IR) in the overweight and obesity population [75]. Although many advantages were presented, the limitations should be considered in our study. The numerous obesity indicators were included to predict the cardio-metabolic risk, but the roles of different obesity indicators in presenting fat mass were unique. BMI presented fat mass in the whole body, while WHR was generally regarded as the index in abdominal fat. The obesity indicators were initially conducted to research the associations with cardiovascular disease and mortality in developed countries. We recruited the experienced indicators to predict cardio-metabolic risk in Chinese adults, which may be the main reason why the ABSI failed to predict cardio-metabolic risk. In addition, the study was conducted with rural populations residing in southeast China, the unique lifestyle may influence the body shape and metabolic indices. Third, although BRI improved quantification of body shape and provided a more accurate estimate of adipose tissue, the calculation of BRI was so complicated that influenced the clinical application. The longitudinal relationship between BRI and cardio-metabolic risk should be examined in future, meanwhile the multi-centers and minority studies are needed to identify the association of obesity indices with more comprehensive metabolic risk factors.

## Conclusions

The present study indicated BRI, WC, BMI, WHR, and WHtR were likely to be optimal indicators for determining the presence of cardio-metabolic risk, especially for high cardio-metabolic risk. BRI was superior and alternative indicator for predicting accumulated cardio-metabolic risk in Chinese adults.

## Supplementary Information

**Additional file 1: Table S1**. The correlation matrix for multiple anthropometric indicators.* WC* waist circumference;* HC* hip circumference;* BMI* body mass index;* WHR* waist-to-hip ratio;* WHtR* waist-to-height ratio;* BAI* body adiposity index;* ABSI* a body shape index;* BRI* body roundness index, *P value < 0.01. **Table S2**. Area under curves (95% CI) of anthropometric indicators to predict cardio-metabolic risk factors. **P value < 0.001, the inter-group difference of AUCs under the ROC curve;* WC* waist circumference;* HC* hip circumference;* BMI* body mass index;* WHR* waist-to-hip ratio;* WHtR* waist-to-height ratio;* BAI* body adiposity index;* ABSI* a body shape index;* BRI* body roundness index,* BP* blood pressure;* FPG* fasting plasma glucose;* TG* triglyceride;* HDL-C* high-density lipoprotein cholesterol;* SUA* serum uric acid.

**Additional file 2: Figure S1**. Heatmap of the correlation between anthropometric indicators. (a) The correlation matrix between anthropometric indicators in males; (b) The correlation matrix between anthropometric indicators in females; *WC* waist circumference; *HC* hip circumference; *BMI* body mass index; *WHR* waist-to-hip ratio; *WHtR* waist-to-height ratio; *BAI* body adiposity index; *ABSI* a body shape index; *BRI* body roundness index.

**Additional file 3: Figure S2**. ROC curves of anthropometric indicators to predict multiple cardiometabolic risk factors. (a) ROC curves of anthropometric indicators to predict elevated BP in male; (b) ROC curves of anthropometric indicators to predict elevated FPG in male; (c) ROC curves of anthropometric indicators to predict elevated TG in male; (d) ROC curves of anthropometric indicators to predict reduced HDL-C in male; (e) ROC curves of anthropometric indicators to predict elevated SUA in male; (f) ROC curves of anthropometric indicators to predict elevated BP in female; (g) ROC curves of anthropometric indicators to predict elevated FPG in female; (h) ROC curves of anthropometric indicators to predict elevated TG in female; (i) ROC curves of anthropometric indicators to predict reduced HDL-C in female; (j) ROC curves of anthropometric indicators to predict elevated SUA in female; *WC* waist circumference; *HC* hip circumference; *BMI* body mass index; *WHR* waist-to-hip ratio; *WHtR* waist-to-height ratio; *BAI* body adiposity index; *ABSI* a body shape index; *BRI* body roundness index, *BP* blood pressure; *FPG* fasting plasma glucose; *TG* triglyceride; *HDL-C* high-density lipoprotein cholesterol; *SUA* serum uric acid.

## Data Availability

The datasets used and/or analyzed during the current study are available from the corresponding author on reasonable request.

## Abbreviations

ROC: Receiver operating characteristic curve
AUC: Areas under the curve
CVD: Cardiovascular disease
T2DM: Type 2 diabetes mellitus
EDTA: Ethylene diamine tetraacetic acid
MetSy: Metabolic syndrome
WC: Waist circumference
HC: Hip circumference
BMI: Body mass index
WHR: Waist-to-hip ratio
WHtR: Waist-to-height ratio
BAI: Body adiposity index
ABSI: A body shape index
BRI: Body roundness index
SBP: Systolic blood pressure
DBP: Diastolic blood pressure
FPG: Fasting plasma glucose
TG: Triglyceride
HDL-C: High-density lipoprotein cholesterol
LDL-C: Low-density lipoprotein cholesterol
SUA: Serum uric acid
BP: Blood press
ALT: Alanine transaminase
AST: Sspartate transaminase
