# Supplementary material for: Body roundness index is a superior indicator to associate with the cardio‐metabolic risk: evidence from a cross‐sectional study with 17,000 Eastern-China adults
Source: BMC Cardiovasc Disord. 2021 Feb 16;21:97. doi: 10.1186/s12872-021-01905-x (PMC7885560; doi:10.1186/s12872-021-01905-x)
Supplement: Supplementary file 1 — Additional file 1: Table S1. The correlation matrix for multiple anthropometric indicators. WC waist circumference; HC hip circumference; BMI body mass index; WHR waist-to-hip ratio; WHtR waist-to-height ratio; BAI body adiposity index; ABSI a body shape index; BRI body roundness index, *P value < 0.01. Table S2. Area under curves (95% CI) of anthropometric indicators to predict cardio-metabolic risk factors. **P value < 0.001, the inter-group difference of AUCs under the ROC curve; WC waist circumference; HC hip circumference; BMI body mass index; WHR waist-to-hip ratio; WHtR waist-to-height ratio; BAI body adiposity index; ABSI a body shape index; BRI body roundness index, BP blood pressure; FPG fasting plasma glucose; TG triglyceride; HDL-C high-density lipoprotein cholesterol; SUA serum uric acid. [file 12872_2021_1905_MOESM1_ESM.docx]

Body roundness index is a superior indicator to associate with the cardio-metabolic risk: Evidence from a cross-sectional study with 17,000 Eastern-China adults

Jinjian Xu^1#^, Liqun Zhang^2#^, Qiong Wu^1#^, Yaohan Zhou^1^, Ziqi Jin^1^, Zhijian Li^4^, Yimin Zhu^1, 3*^

^1^Department of Epidemiology & Biostatistics, School of Public Health, Zhejiang University, Hangzhou 310058, Zhejiang, China

^2^Putuo District People's Hospital, Zhoushan 316100, Zhejiang, China

^3^Department of Respiratory, Sir Run Run Shaw Hospital Affiliated to School of Medicine, Zhejiang University, Hangzhou 310020, Zhejiang, China

^4^The First Affiliated Hospital, School of Medicine, Zhejiang University, Hangzhou, 310058, Zhejiang, China

^#^Equal contribution

*Corresponding author: Yimin Zhu, MD, PhD. E-mail address: [zhuym@zju.edu.cn](mailto:zhuym@zju.edu.cn)

Additional Table 1 The correlation matrix for multiple anthropometric indicators

|  |  | Males | | | | | | | | | | |
| --- | --- | --- | --- | --- | --- | --- | --- | --- | --- | --- | --- | --- |
|  |  | Height | Weight | WC | HC | BMI | WHR | WHtR | BAI | ABSI | BRI |  |
| Females | Height | 1 | 0.51* | 0.19* | 0.32* | 0.01 | -0.03* | -0.18* | -0.5* | -0.02 | -0.18* | Height |
|  | Weight | 0.45* | 1 | 0.79* | 0.80* | 0.86* | 0.44* | 0.60* | 0.31* | -0.02 | 0.60* | Weight |
|  | WC | 0.07* | 0.74* | 1 | 0.80* | 0.80* | 0.78* | 0.93* | 0.57* | 0.54* | 0.93* | WC |
|  | HC | 0.27* | 0.79* | 0.74* | 1 | 0.74* | 0.25* | 0.68* | 0.65* | 0.23* | 0.68* | HC |
|  | BMI | -0.05* | 0.87* | 0.78* | 0.74* | 1 | 0.53* | 0.80* | 0.67* | -0.02 | 0.80* | BMI |
|  | WHR | -0.14* | 0.35* | 0.78* | 0.16* | 0.47* | 1 | 0.79* | 0.24* | 0.62* | 0.78* | WHR |
|  | WHtR | -0.25* | 0.57* | 0.95* | 0.63* | 0.77* | 0.80* | 1 | 0.75* | 0.55* | 0.99* | WHtR |
|  | BAI | -0.48* | 0.39* | 0.62* | 0.71* | 0.71* | 0.24* | 0.76* | 1 | 0.22* | 0.76* | BAI |
|  | ABSI | -0.10* | -0.02 | 0.61* | 0.19* | 0.02 | 0.71* | 0.63* | 0.24* | 1 | 0.54* | ABSI |
|  | BRI | -0.25* | 0.57* | 0.94* | 0.63* | 0.77* | 0.79* | 0.99* | 0.76* | 0.62* | 1 | BRI |

*WC* waist circumference; *HC* hip circumference; *BMI* body mass index; *WHR* waist-to-hip ratio; *WHtR* waist-to-height ratio; *BAI* body adiposity index; *ABSI* a body shape index; *BRI* body roundness index, **P* value < 0.01

Additional Table 2 Area under curves (95% CI) of anthropometric indicators to predict cardio-metabolic risk factors

|  | WC | BMI | WHR | WHtR | BAI | ABSI | BRI |
| --- | --- | --- | --- | --- | --- | --- | --- |
| Male |  |  |  |  |  |  |  |
| Elevated BP | 0.62 (0.61-0.63) ^**^ | 0.62 (0.61-0.63) ^**^ | 0.63 (0.61-0.64) ^**^ | 0.64 (0.63-0.65) ^**^ | 0.61 (0.59-0.62) ^**^ | 0.58 (0.56-0.59) ^**^ | 0.64 (0.63-0.65) ^**^ |
| Elevated FPG | 0.61 (0.59-0.62) ^**^ | 0.62 (0.6-0.64) ^**^ | 0.60 (0.58-0.61) ^**^ | 0.61 (0.60-0.63) ^**^ | 0.59 (0.57-0.61) ^**^ | 0.53 (0.51-0.55) ^**^ | 0.61 (0.60-0.63) ^**^ |
| Elevated TG | 0.70 (0.69-0.72) ^**^ | 0.70 (0.69-0.72) ^**^ | 0.68 (0.66-0.69) ^**^ | 0.70 (0.68-0.71) ^**^ | 0.62 (0.61-0.64) ^**^ | 0.58 (0.57-0.60) ^**^ | 0.70 (0.68-0.71) ^**^ |
| Reduced HDL-C | 0.64 (0.62-0.66) ^**^ | 0.64 (0.63-0.66) ^**^ | 0.62 (0.61-0.64) ^**^ | 0.63 (0.61-0.65) ^**^ | 0.57 (0.55-0.58) ^**^ | 0.54 (0.52-0.56) ^**^ | 0.63 (0.61-0.65) ^**^ |
| Elevated SUA | 0.62 (0.60-0.63) ^**^ | 0.62 (0.61-0.64) ^**^ | 0.61 (0.59-0.62) ^**^ | 0.61 (0.60-0.63) ^**^ | 0.56 (0.55-0.58) ^**^ | 0.54 (0.52-0.55) ^**^ | 0.61 (0.60-0.63) ^**^ |
| Female |  |  |  |  |  |  |  |
| Elevated BP | 0.65 (0.64-0.66) ^**^ | 0.63 (0.62-0.64) ^**^ | 0.66 (0.65-0.67) ^**^ | 0.67 (0.66-0.68) ^**^ | 0.63 (0.62-0.64) ^**^ | 0.62 (0.61-0.63) ^**^ | 0.67 (0.66-0.68) ^**^ |
| Elevated FPG | 0.61 (0.60-0.63) ^**^ | 0.61 (0.59-0.62) ^**^ | 0.58 (0.56-0.59) ^**^ | 0.61 (0.59-0.62) ^**^ | 0.58 (0.57-0.60) ^**^ | 0.55 (0.53-0.56) ^**^ | 0.61 (0.59-0.62) ^**^ |
| Elevated TG | 0.69 (0.68-0.70) ^**^ | 0.68 (0.67-0.69) ^**^ | 0.67 (0.66-0.68) ^**^ | 0.69 (0.68-0.70) ^**^ | 0.63 (0.62-0.64) ^**^ | 0.61 (0.60-0.62) ^**^ | 0.69 (0.68-0.70) ^**^ |
| Reduced HDL-C | 0.62 (0.61-0.63) ^**^ | 0.62 (0.61-0.63) ^**^ | 0.63 (0.62-0.64) ^**^ | 0.62 (0.61-0.63) ^**^ | 0.55 (0.54-0.57) ^**^ | 0.55 (0.54-0.56) ^**^ | 0.62 (0.61-0.63) ^**^ |
| Elevated SUA | 0.69 (0.66-0.72) ^**^ | 0.68 (0.66-0.71) ^**^ | 0.66 (0.63-0.69) ^**^ | 0.70 (0.68-0.73) ^**^ | 0.66 (0.63-0.69) ^**^ | 0.60 (0.58-0.63) ^**^ | 0.70 (0.68-0.73) ^**^ |

***P* value < 0.001, the inter-group difference of AUCs under the ROC curve; *WC* waist circumference; *HC* hip circumference; *BMI* body mass index; *WHR* waist-to-hip ratio; *WHtR* waist-to-height ratio; *BAI* body adiposity index; *ABSI* a body shape index; *BRI* body roundness index, *BP* blood pressure; *FPG* fasting plasma glucose; *TG* triglyceride; *HDL-C* high-density lipoprotein cholesterol; *SUA* serum uric acid

Additional Figure 1 Heatmap of the correlation between anthropometric indicators

(a) The correlation matrix between anthropometric indicators in males; (b) The correlation matrix between anthropometric indicators in females; *WC* waist circumference; *HC* hip circumference; *BMI* body mass index; *WHR* waist-to-hip ratio; *WHtR* waist-to-height ratio; *BAI* body adiposity index; *ABSI* a body shape index; *BRI* body roundness index

Additional Figure 2 ROC curves of anthropometric indicators to predict multiple cardiometabolic risk factors

(a) ROC curves of anthropometric indicators to predict elevated BP in male; (b) ROC curves of anthropometric indicators to predict elevated FPG in male; (c) ROC curves of anthropometric indicators to predict elevated TG in male; (d) ROC curves of anthropometric indicators to predict reduced HDL-C in male; (e) ROC curves of anthropometric indicators to predict elevated SUA in male; (f) ROC curves of anthropometric indicators to predict elevated BP in female; (g) ROC curves of anthropometric indicators to predict elevated FPG in female; (h) ROC curves of anthropometric indicators to predict elevated TG in female; (i) ROC curves of anthropometric indicators to predict reduced HDL-C in female; (j) ROC curves of anthropometric indicators to predict elevated SUA in female; *WC* waist circumference; *HC* hip circumference; *BMI* body mass index; *WHR* waist-to-hip ratio; *WHtR* waist-to-height ratio; *BAI* body adiposity index; *ABSI* a body shape index; *BRI* body roundness index, *BP* blood pressure; *FPG* fasting plasma glucose; *TG* triglyceride; *HDL-C* high-density lipoprotein cholesterol; *SUA* serum uric acid
