# Supplementary figures and images for: Body roundness index is a superior indicator to associate with the cardio‐metabolic risk: evidence from a cross‐sectional study with 17,000 Eastern-China adults
Source: BMC Cardiovasc Disord. 2021 Feb 16;21:97. doi: 10.1186/s12872-021-01905-x (PMC7885560; doi:10.1186/s12872-021-01905-x)

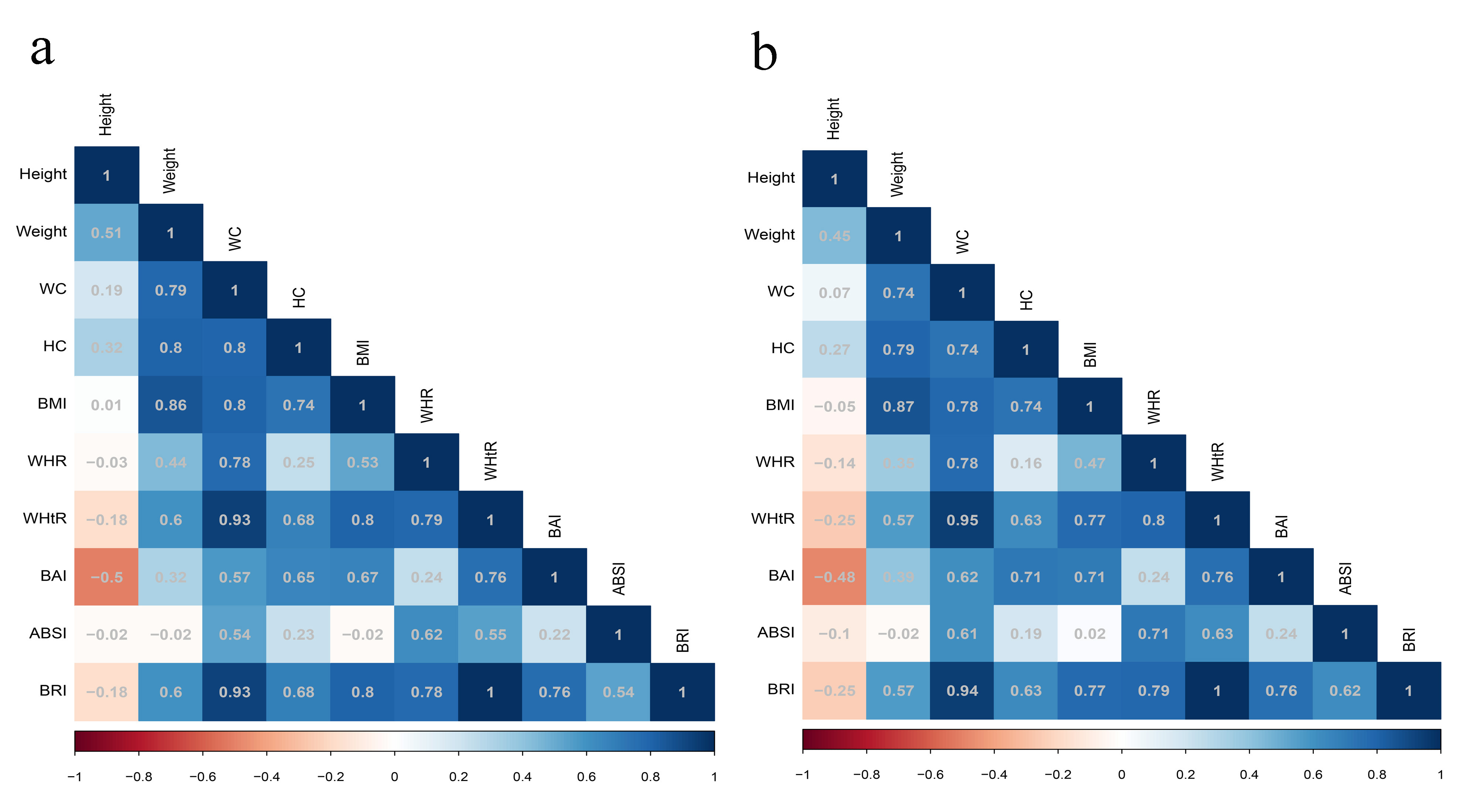

Supplement: Supplementary file 2 — Additional file 2: Figure S1. Heatmap of the correlation between anthropometric indicators. (a) The correlation matrix between anthropometric indicators in males; (b) The correlation matrix between anthropometric indicators in females; WC waist circumference; HC hip circumference; BMI body mass index; WHR waist-to-hip ratio; WHtR waist-to-height ratio; BAI body adiposity index; ABSI a body shape index; BRI body roundness index. [file 12872_2021_1905_MOESM2_ESM.jpg]
